# Genetic Relatedness of Infectious Hypodermal and Hematopoietic Necrosis Virus Isolates, United States, 2019

**DOI:** 10.3201/eid2802.211874

**Published:** 2022-02

**Authors:** Arun K. Dhar, Roberto Cruz-Flores, Janet Warg, Mary L. Killian, Andrew Orry, Jorge Ramos, Michelle Garfias, Gregory Lyons

**Affiliations:** The University of Arizona, Tucson, Arizona, USA (A.K. Dhar, R. Cruz-Flores, J. Ramos, M. Garfias, G. Lyons);; US Department of Agriculture National Veterinary Services Laboratories, Ames, Iowa, USA (J. Warg, M.L. Killian);; Molsoft, Inc., San Diego, California, USA (A. Orry)

**Keywords:** infectious hypodermal and hematopoietic necrosis virus, IHHNV, *Decapod penstylhamaparvovirus* 1, United States, shrimp, viruses, *Penaeus vannamei*, densovirus, parvovirus

## Abstract

Infectious hypodermal and hematopoietic necrosis virus (IHHNV) is a nonenveloped, linear, single-stranded DNA virus belonging to the family *Parvoviridae* and is a World Organisation for Animal Health (OIE)–notifiable crustacean pathogen. During screening of *Penaeus vannamei* shrimp from 3 commercial shrimp facilities in the United States for a panel of OIE-listed (n = 7) and nonlisted (n = 2) crustacean diseases, shrimp from these facilities tested positive for IHHNV. Nucleotide sequences of PCR amplicons showed 99%–100% similarity to IHHNV isolates from Latin America and Asia. The whole genome of the isolates also showed high similarity to type 2 infectious forms of IHHNV. Phylogenetic analysis using capsid gene and whole-genome sequences demonstrated that the isolates clustered with an IHHNV isolate from Ecuador. The detection of an OIE-listed crustacean pathogen in the United States highlights the need for biosecurity protocols in hatcheries and grow-out ponds to mitigate losses.

The *Penstylhamaparvovirus* species *Decapod penstylhamaparvovirus 1*, commonly known as infectious hypodermal and hematopoietic necrosis virus (IHHNV), is the smallest of known shrimp viruses belonging to the family *Parvoviridae*, subfamily *Hamaparvovirinae* (*1*,*2*). The virions are icosahedral, nonenveloped, measure 22–23 nm in size, and contain a single-stranded DNA genome ≈4.1 kb in length (*3*,*4*).

IHHNV was first reported in juveniles and subadults of blue shrimp (*Penaeus stylirostris*) in Hawaii in the early 1980s and caused mass deaths (*5*). The virus also caused large-scale deaths in blue shrimp in Mexico (*6*). The virus outbreak in *P. stylirostris* shrimp in Mexico in the early 1980s led to a transition in the captive breeding program from a more susceptible and physically larger shrimp species, *P. stylirostris* (a preferred cultured species in the mid-1980s), to the smaller but more tolerant *P. vannamei* shrimp (*7*). In the black tiger shrimp (*P. monodon*), another economically vital species, IHHNV is reported to cause asymptomatic infection and deformities (*4*,*8*). In a recent study, a farm-level IHHNV infection resulted in reduced growth performance, a higher food-conversion ratio, and a lower survival rate, which led to a production loss of approximately US $67,000 per hectare of farm gate value when ponds were stocked with IHHNV-infected postlarvae having high viral load compared with postlarvae with a low viral load (*9*). This finding suggests that IHHNV remains an economically relevant viral pathogen in shrimp aquaculture.

Most of the genetic lines of *P. vannamei* shrimp farmed in Latin America and Asia today are claimed to be tolerant or resistant to the virus, and IHHNV is assumed to have no ill effect on the production parameters in these shrimp lines, although scientific evidence to support this assumption is lacking. Considering these genetic lines can tolerate high levels of IHHNV without displaying clinical manifestations of the disease, transboundary movement of shrimp broodstock and larvae from these lines might lead to widespread distribution of IHHNV unless rigorous biosecurity is practiced in hatcheries. Routine disease surveillance that adheres to the US Department of Agriculture and World Organisation for Animal Health (OIE) guidelines and diagnostics BLAST performed by the Aquaculture Pathology Laboratory (APL) and the National Veterinary Services Laboratories (NVSL) have helped to mitigate the spread of major pathogens in the United States. This success is reflected by the 26-year absence of the virus from all US-based farms until now.

During June 2019, *P. vannamei* shrimp samples from broodstock and postlarvae originating in commercial shrimp facilities in Texas and Florida were submitted to the APL at the University of Arizona for routine screening for viral, bacterial, and fungal pathogens. IHHNV was the only pathogen identified in shrimp from both facilities. Trace testing resulted in additional IHHNV-positive animals. We further characterized the IHHNV strains detected in the United States by genome sequencing and determined their genetic similarities to other IHHNV strains. Considering the history of economic losses caused by IHHNV over the past 4 decades, the recent detection of IHHNV in commercial facilities in the United States highlights the need to enhance biosecurity to prevent spread and future disease outbreaks caused by IHHNV or other diseases in shrimp aquaculture. The need to further enhance diagnostic testing and biosecurity measures was underscored by the subsequent detection of IHNNV in the United Kingdom and Canada, which was directly linked to commercial shrimp facilities in the United States (*10*,*11*). The objectives of this study were to elucidate the genomic characteristics of the virus to ensure that the IHHNV isolate is an infectious form of the virus and not a genome-integrated form, as well as to determine its genetic relatedness to IHHNV isolates described in the literature. 

## Materials and Methods

### Sample Submission

Samples of *P. vannamei* shrimp were collected from commercial shrimp facilities, 2 in Texas (case nos. 19-428 and 19-644) and 1 in Florida (case no. 19-490) and submitted to the University of Arizona APL. Samples from case no. 19–428 consisted of 6 vials containing shrimp pleopods and postlarvae preserved in ethanol. Case no. 19-644 consisted of 4 bags of frozen whole shrimp. Case no. 19-490 consisted of pleopods in ethanol. We collected representative samples (≈30 mg) for nucleic acid extraction. Samples testing positive for IHHNV at the University of Arizona were submitted to the NVSL (Ames, Iowa, USA) for confirmation and further characterization.

### Nucleic Acid Extraction and PCR

We extracted total nucleic acid from pools of pleopods and whole postlarvae (case nos. 19-428 and 19-490). For case no. 19-644, in which whole animals were submitted, we extracted total nucleic acid from tail muscle tissues (for screening systemic pathogens) or hepatopancreas (for screening enteric pathogens) by using the Maxwell 16 Cell LEV DNA and RNA Purification Kits (Promega, https://www.promega.com) or using the Maxwell 16 Instrument configured with LEV Hardware, according to manufacturer instructions.

We used pleopod DNA to screen for IHHNV (*12*), IHHNV–related genome-integrated sequence (*13*), and white spot syndrome virus (*14*). We used hepatopancreas DNA for PCR screening of *Baculovirus penaei* (according to an in-house method), necrotizing hepatopancreatitis bacterium (*15*), *Enterocytozoon hepatopenaei* (*16*), and acute hepatopancreatic necrosis disease (*17*). We evaluated pleopod and tail muscle RNA by using reverse transcription PCR tests for yellow head virus (*18*), Taura syndrome virus (*19*), and infectious myonecrosis virus (*20*). For all reactions, we used 1 µL of nucleic acids at a concentration range of 1–50 ng/µL.

We performed testing for IHHNV first by using the OIE-recommended 389 F/R primer pair, followed by the 309 F/R primer pair to detect the presence of the infectious form of IHHNV (*13*). We visualized PCR products on a 1.5% agarose gel to confirm presence of viral DNA. We used DNA from samples testing positive for IHHNV by PCR screening to amplify the nonstructural (NS1) and capsid protein (CP) genes. We amplified the NS1 gene by using 2 sets of primers to generate 2 amplicons with a 66-bp overlap (Table 1) and the CP gene by using the method of Robles-Sikisaka et al. (*21*), which yields a 1,088-bp amplicon.

**Table 1:** Primers used for the detection of the IHHNV genome in commercially raised *Penaeus vannamei* shrimp, Texas and Florida, United States, 2019*

Target gene	Primer name	Sequence, 5′ → 3′	Amplicon size, bp	Reference
NS1	389F	CGGAACACAACCCGACTTTA	389	(*13*)
NS1	389R	GGCCAAGACCAAAATACGAA		
NS1	IHHNV-309F	TCCAACACTTAGTCAAAACCAA	309	(*13*)
NS1	IHHNV-309R	TGTCTGCTACGATGATTATCCA		
NS1 (Left)	159_IH_NS1_S	AACTGACGAGTGAAGAGGCT	1295	This study
NS1 (Left)	1446_IH_NS1_AS	GTGTCCGGAGTATGTGATGT		This study
NS1 (Right)	1400_IH_NS1_S	GGACGAACGCCAAACTTCAC	1313	This study
NS1 (Right)	2698_IH_NS1_AS	ATCTGTGTGGGTCTGGTCC		This study
CP	CP1F	GATCACCAGCACGACTTCCT	1088	(*21*)
CP	CP2R	CGGGTATATATTGCACATCGAA		

*CP, capsid protein gene; IHHNV, infectious hypodermal and hematopoietic necrosis virus; NS1, nonstructural gene 1.

### Sanger Sequencing and Data Analysis

We column purified PCR amplicons by using the GeneJET PCR Purification Kit (ThermoFisher Scientific, https://www.thermofisher.com) according to manufacturer instructions. Purified PCR products were submitted to the University of Arizona Genetics Core for Sanger sequencing (Table 2). We aligned sequence data in Geneious Prime to generate a consensus sequence (*22*) and compared those sequences with published genomes using BLASTn (*23*).

**Table 2:** Additional primers designed and used for Sanger sequencing of CP and NS1 genes of IHHNV, Texas and Florida, United States, 2019*

Target gene	Primer name	Sequence, 5′ → 3′
NS1	159_IH_NS1_S	AAC TGA CGA GTG AAG AGG CT
NS1	1446_IH_NS1_AS	GTG TCC GGA GTA TGT GAT GT
NS1	482_IHHNV_NS1_S	CCC CAA CAA ATA TCG CTG CG
NS1	511_IHHNV_NS1_S	AGA TCA CAT TCT ACC GTG GTG
NS1	342_IH_NS1_Seq_AS	ACTT GTA CTT ACA TTT GTA T
NS1	1400_IH_NS1_S	GGA CGA ACG CCA AAC TTC AC
NS1	2627_IH_NS1_Seq_S	CAA GCC CAA GGA AAA GAT CC
NS1	2390 CP_S	CTA CTG GGT ACC ACC AGC
NS1	2698 IH_NS1_AS	ATC TGT GTG GGT CTG GTC C
NS1	2540 IH_CP_S	AGG CCT CTT CCA AGA ATA CG
NS1	2682 IHHNV_NS1_AS	ACT TGA TCC TTC GGC GTG TT
NS1	2540 IH_CP_S	AGG CCT CTT CCA AGA ATA CG
NS1	2682 IHHNV_NS1_AS	ACT TGA TCC TTC GGC GTG TT
NS1	IHHNV-1942_F	GTC ACT AAT TAC AAA CCT GCA G
NS1	IHHNV-2020R	GCA TAT TGT CGT AGT CTG GT
CP	IHHNV-CP_S	ATG TGC GCC GAT TCA ACA AG
CP	IH_CP_Seq_S	CAT AAT CAA CTA TCA ACT AA
CP	IH_CP_Seq_AS	TGC CAA TGT TAC GTC GGT TTC C

*CP, capsid protein gene; IHHNV, infectious hypodermal and hematopoietic necrosis virus; NS1, nonstructural gene 1.

### Whole-Genome Sequencing and Bioinformatics Analyses

We extracted total nucleic acids from tissue homogenates for initial testing by using the MagMAX-Ambion kit 1836 on an automated MagMAX Express magnetic particle processor (ThermoFisher Scientific). We extracted additional DNA from tissue homogenates treated with Benzonase to reduce host DNA by using the DNeasy Blood and Tissue Kit (QIAGEN, https://www.quiagen.com). We prepared libraries by using the Ion Xpress Plus Fragment Library Kit (ThermoFisher Scientific) according to manufacturer instructions and templates by using the Ion 520 on the Ion Chef System, then sequenced by using an Ion 520 Chip on the Ion S5 System (ThermoFisher Scientific). We performed reference-based mapping (GenBank accession no. NC_002190) by using SeqMan NGen version 14 (DNASTAR, https://www.dnastar.com) with default parameters for automatic read trimming and assembly and verified alignments resulting in the final consensus sequence. The genomes (minus ends because random priming was used) of IHHNV from Texas and Florida were deposited in GenBank (accession nos. MN968717.1 and MN968716.1).

### Phylogenetic Analyses

Initially, we inferred the phylogenetic analysis by using the neighbor-joining method (*24*) for the CP gene of IHHNV with 1,000 bootstraps. The tree was drawn to scale; branch lengths were in the same units as those of the evolutionary distances used to infer the phylogenetic tree. We computed the evolutionary distances (base substitutions per site) by using the maximum composite likelihood method (*25*). This analysis involved 32 nt sequences. All ambiguous positions were removed for each sequence pair (pairwise deletion option). A total of 995 positions were represented in the final dataset. We conducted evolutionary analyses in MEGA X (*25*).

We downloaded all available complete genomes from GenBank on November 1, 2019, for phylogenetic analysis. We inferred evolutionary history by using the maximum-likelihood method based on the Tamura-Nei model (*24*). We obtained initial tree(s) for the heuristic search automatically by applying neighbor-joining and BioNJ algorithms to a matrix of pairwise distances estimated by using the maximum-likelihood approach and then by selecting the topology with superior log likelihood value. The tree is drawn to scale; branch lengths are measured in the number of substitutions per site. The analysis involved 28 nt sequences. Codon positions included were 1st+2nd+3rd+Noncoding. We eliminated all positions containing gaps and missing data. A total of 2,368 positions were represented in the final dataset. We conducted evolutionary analyses in MEGA7 (*25*).

### Predicted Tertiary Structure of IHHNV CP

We performed all molecular modeling by using ICM-Pro desktop modeling software version 3.8 (MolSoft LLC, https://www.molsoft.com). Homology models of the CP gene of the IHHNV Texas isolate (case no. 19-428) and Florida isolate (case no. 19-490) were built by using the crystal structure of *Decapod penstylhamaparvovirus* 1 CP as a template (PDB code 3N7X) (*26*). First, we built a sequence alignment that demonstrated the amino acid sequences have very high homology (88%). Considering that the Texas isolates (case nos. 19-428 and 19-644) had identical amino acid sequences, we used only 1 for the analysis. The amino acid sequence of the isolates was then threaded onto the backbone of the full biomolecule capsid template structure by using ICM-Pro, and the model was energy minimized.

## Results

### Detection of IHHNV in Shrimp Hatchery– and Field-Collected Samples from Texas and Florida

**Figure 1:**
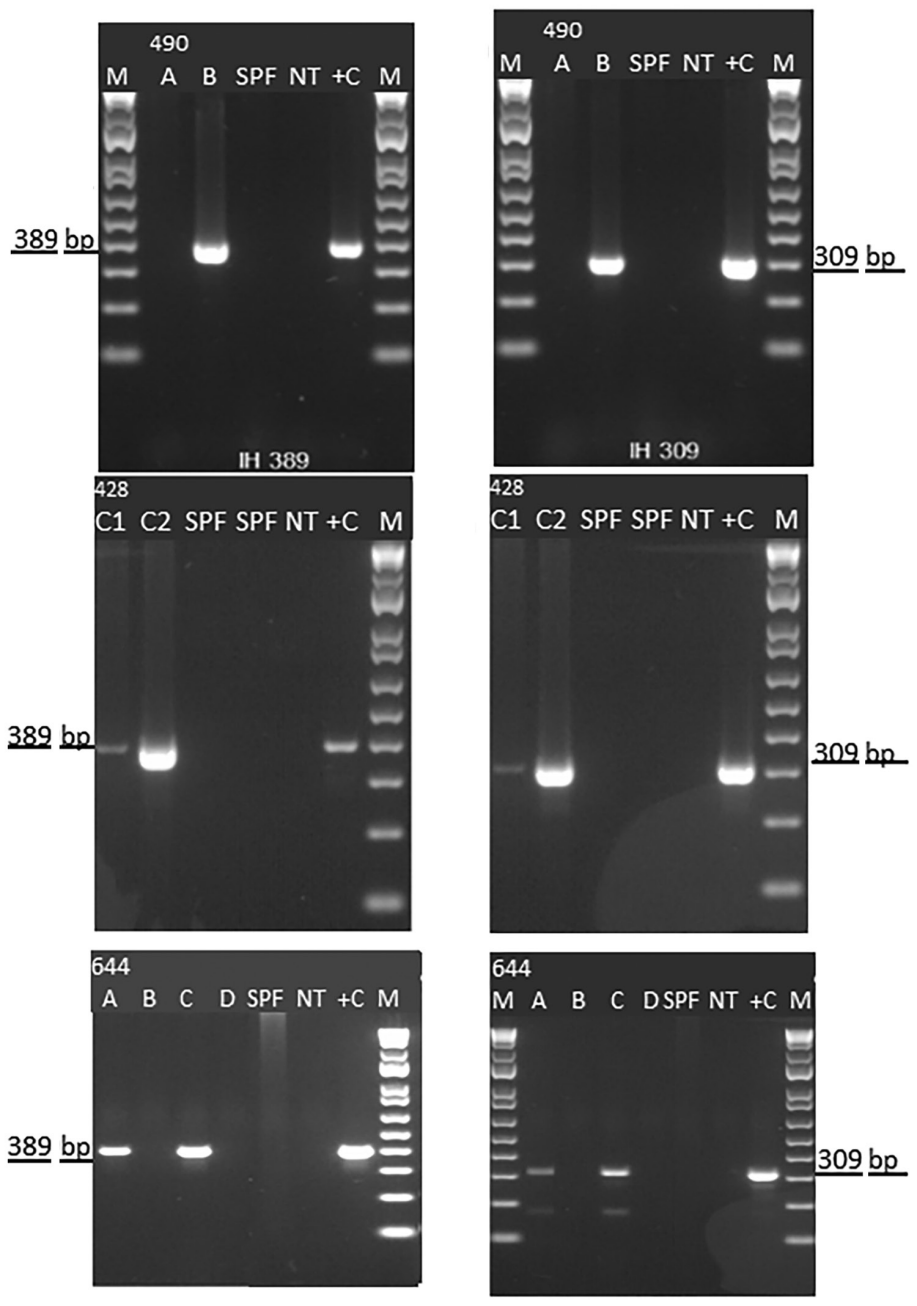
Detection of infectious hypodermal and hematopoietic necrosis virus (IHHNV) in *Penaeus vannamei* shrimp from the United States by conventional PCR, 2019. Agarose gel photographs show 389-bp IHHNV–specific amplicon (left column) and 309-bp IHHNV–specific amplicon (right column). Top row represents case number 19-490, *P. vannamei* broodstock samples originating in Florida (A and B). Middle row represents case number 19-428, *P. vannamei* post-larvae samples originating in Texas (C1 and C2). Bottom row represents case number 19-644, frozen *P. vannamei* shrimp originating from an indoor farm in Texas (A, B, C, and D). Lane M, 100-bp molecular weight marker (New England Biolabs, Inc., https://www.neb.com); lane SPF, specific pathogen-free *P. vannamei* shrimp; lane NT, no template control; lane +C, positive control for PCR.

We detected IHHNV in samples from Texas (case nos. 19-428 and 19-644) and Florida (case no. 19-490) by using the OIE-recommended conventional PCR-based diagnostic method (*12*) (Figure 1). These results confirmed the first detection of the virus in 2 commercial aquaculture facilities in the United States since 1993 (*27*).

### Amplification and Sequencing of CP and NS1 Genes of IHHNV

The complete CP and NS1 genes of the IHHNV isolates from Texas and Florida were successfully amplified in overlapping fragments. The 2 amplicons together represent ≈88% of total length of IHHNV genome. We sequenced the NS1 and CP amplicons of both Texas and Florida IHHNV isolates. BLAST analysis demonstrated that both genes showed highest similarity to homologous genes of the IHHNV Ecuador strain (GenBank accession no. AY362548.1) (Table 3).

**Table 3:** Sequence similarity of CP and NS-1 genes of Texas and Florida IHHNV isolates to IHHNV Ecuador strain, 2019*

Case no.	CP identity, %	NS1 identity, %	Strain	GenBank accession no.
19–428-C2	99.36	99.06	Ecuador	AY362548.1
16–490-B	99.72	99.69	Ecuador	AY362548.1
19–644-A	99.36	99.65	Ecuador	AY362548.1
19–644-C	99.36	99.65	Ecuador	AY362548.1

*CP, capsid protein gene; IHHNV, infectious hypodermal and hematopoietic necrosis virus; NS1, nonstructural gene 1.

### Whole-Genome Sequencing of IHHNV Texas and Florida Isolates

For isolate 19-490 from Florida, 22,166 sequences were assembled to generate a consensus sequence 3749 bp in length with a median depth of coverage of 1,036×. For isolates 19-428 and 19-644 from Texas, 118,348 sequences were assembled to generate a consensus sequence of 3,750 bp in length with a median depth of coverage of 5,825×. Consensus sequences represent the complete coding region of the virus. The 2 isolates shared 99.71% sequence identity. Furthermore, the sequences had 99.44% (Texas) and 99.52% (Florida) identity to IHHNV from Ecuador.

### Phylogenetic Analysis

**Figure 2:**
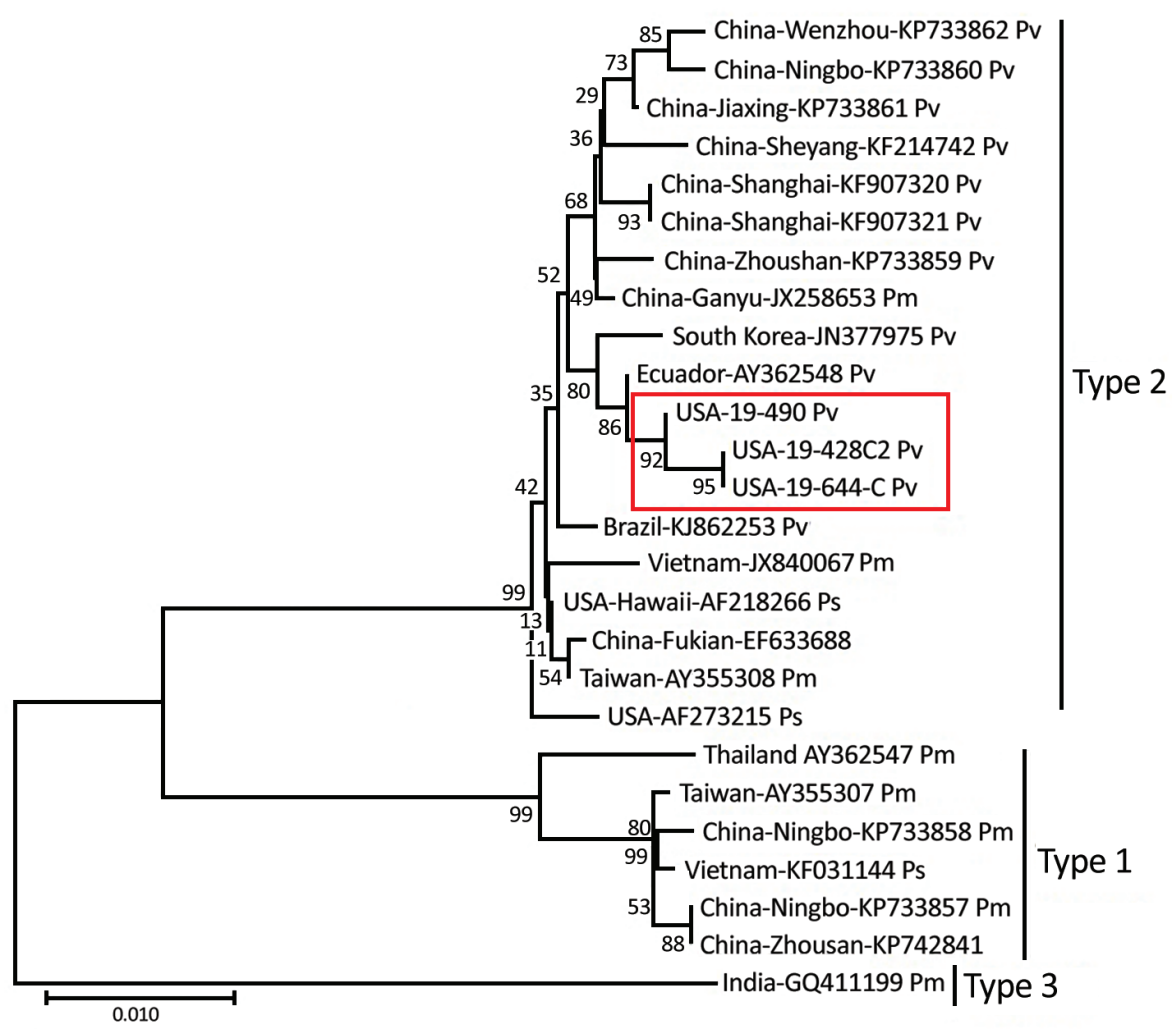
Evolutionary relationships of the infectious hypodermal and hematopoietic necrosis virus (IHHNV) strains (19-428, 19-490, and 19-644) recently detected in the United States and published capsid protein gene sequences. The recent IHHNV strains (red box) fall into the type 2 lineage. The evolutionary history was inferred by using the neighbor-joining method (*24*). The optimal tree with the sum of branch length = 0.20086053 is shown. The percentage of replicate trees in which the associated taxa clustered together in the bootstrap test (1,000 replicates) are shown next to the branches. The tree is drawn to scale, with branch lengths in the same units as those of the evolutionary distances used to infer the phylogenetic tree. The evolutionary distances were computed by using the maximum-likelihood method (*25*). Based upon full-genome phylogenetic analysis, the Texas and Florida IHHNV viruses appear to be related to a strain from Ecuador (GenBank accession no. AY362548.1). Scale bar indicates substitutions per site.

Phylogenetic analysis using only the CP gene sequences showed that IHHNV from both Texas and Florida formed a highly supported cluster with the Ecuador strain of IHHNV. This cluster represents the type 2 infectious form of IHHNV (Figure 2). The IHHNV in this cluster infect penaeid species including *P. vannamei*, *P. stylirostris*, and *P. monodon* shrimp. In the neighbor-joining phylogenetic tree, the type 3 clade is represented by IHHNV isolates from India, Tanzania, Madagascar, and Australia. These strains infect *P. monodon* shrimp, are noninfectious, and remain integrated into the *P. monodon* genome. The third genotype, type 1, is represented by isolates from the Philippines and Thailand that infect both *P. monodon* and *P. vannamei* shrimp. Whole-genome phylogeny showed equivalent results (tree not shown).

### Predicted Tertiary Structure of CP of Texas and Florida IHHNV Isolates

**Figure 3:**
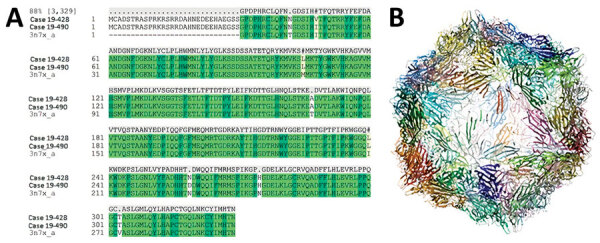
Alignment and structure of infectious hypodermal and hematopoietic necrosis virus (IHHNV) strains recently detected in Texas (19-428) and Florida (19-490), USA. A) Multiple alignment of amino acid sequence based on translation of the capsid protein gene of isolates with *Penaeus stylirostris densovirus* capsid protein sequence (PDB code 3N7X). B) Predicted tertiary structure of the isolates from Texas and Florida.

The IHHNV Texas and Florida viruses detected have ≈88% amino acid sequence identity with the crystal structure of *Penaeus stylirostris densovirus* (*Pst*DNV) capsid (PDB code 3N7X) (Figure 3, panel A). The crystal structure of *Pst*DNV was determined to 2.5-Å resolution by x-ray crystallography and based on 299 out of 329 aa of capsid protein missed 30 N′-terminal aa (*26*) (Figure 3, panel B). Because of the high sequence similarity, the modeled structure showed key β-barrel motifs that are hallmarks of many icosahedral viruses.

## Discussion

During routine disease surveillance, 3 Pacific white shrimp (*P. vannamei*) samples from 2 facilities in Texas and 1 facility in Florida tested positive for IHHNV. The samples were initially screened according to the OIE-recommended PCR protocol using the primer pair IHHNV 389 F/R (*13*). The results were further confirmed by using primer pair IHHNV 309 F/R. The amplicons 389 bp and 309 bp from all 3 cases were sequenced by Sanger methods; the nucleotide sequence of these amplicons showed a 99% identity with previously reported IHHNV sequences deposited in GenBank. Confirming the presence of IHHV by PCR and genome sequencing is needed for diagnostic certainty, because genome-integrated sequences of IHHNV have been shown to be present in *P. monodon* and *P. vannamei* shrimp (*13*,*28*). Because IHHNV is an OIE-listed disease, once the virus was detected at the APL, an aliquot of all the nucleic acid and tissue samples was sent to the NVSL (Ames, Iowa, USA) for confirmation and further characterization. Under this testing scheme performed by APL and NVSL, IHHNV has been successfully kept at bay from US-based broodstock and hatchery operations for more than 2 decades. This recent detection, however, shows that stringent surveillance is needed to ensure facilities are free of disease before broodstock and larvae are moved across countries and continents. 

The IHHNV genome contains 2 overlapping open reading frames encoding the nonstructural proteins NS1 and NS2 and a third open reading frame that encodes the viral capsid protein (*3*,*4*,*7*). Together, these 3 gene segments comprise ≈88% of the complete genome. In this study, after IHHNV was detected in the 3 samples, the CP (990 bp) and NS1 (2440 bp) genes were amplified and sequenced. Subsequently, the near full-length genome of 2 isolates originating from 2 shrimp facilities in Texas and Florida were sequenced by using next-generation sequencing, generating contigs of 3,750 bases in length for the Texas isolates and 3,749 bases in length for the Florida isolates. All 3 cases (19-428, 19-490, and 19-644) had high sequence identity (99%) among them, as well as to an IHHNV isolate originating in Ecuador (GenBank accession no. AY362548.1). In 2003, two genotypes of IHHNV were described. At that time, type 1 strains were found in the Americas and East Asia (primarily the Philippines), and type 2 strains were detected in Southeast Asia (*29*). Since then, an additional genotype, type 3, has been described, and the geographic range of type 1 and type 2 viruses has spread worldwide. Phylogenetic analysis using the CP gene and full-length genomes indicate the recently detected IHHNV viruses from the United States form a strongly supported cluster with type 2 strains from the Americas. These strains represent the infectious forms of IHHNV and are clearly distinct from type 1 (infectious) and type 3 (noninfectious). (Figure 2). Since the virus was first described in the early 1980s, <50 complete genomes have been published in GenBank, and many of the genomes have incomplete metadata, including date of isolation or detection, which confounds interpretation of phylogenetic analysis. The lack of these critical pieces of information limits the ability to infer transmission of the virus on the basis of phylogeny alone. Epidemiologic information for the Texas isolates and the phylogenetic analysis suggest a link between the detections of IHHNV in Texas and Florida, supporting their close genetic relationship.

The close similarity at the whole-genome level and the phylogenetic data indicate that all sequences derive from a strain from Ecuador, suggesting a common origin for both Texas and Florida strains. This occurrence is not uncommon in shrimp aquaculture; other viral pathogens such as Taura syndrome virus and infectious myonecrosis virus have been traced back to their original source (*30*,*31*). The transboundary movement of shrimp has gone hand-in-hand with the expansion of shrimp farming and will not cease anytime soon. Investment in newer, more sensitive diagnostic methodologies based on CRISPR, digital droplet PCR, and next-generation sequencing approaches could help limit the spread of pathogens (*32*,*33*). In addition, rigorous screening of animals for IHHNV and other pathogens is necessary to make moving shrimp safer and thereby make the industry more sustainable and resilient in the long run. This rigorous screening would entail collaborative efforts between shrimp producers, diagnostic and research institutions, and corresponding government entities.

To determine whether the predicted amino acid sequences of the IHHNV capsid protein from Texas and Florida isolates can conform an icosahedral symmetry, homology modeling was performed with a reference tertiary structure of another IHHNV isolate, *Pst*HPV-1, for which a crystal structure is available in the Protein Data Bank (PDB code 3N7X). Although a sequence identity of 88% between the reference strains and US strains might seem low, this identity is because of the absence of 20 aa from the N′-terminal of the reference strain. The analysis of the same sections (excluding the first 20 aa from the N′-terminal of US strains) of the CP of the 3 strains demonstrates a sequence identity of 97.5%. The tertiary structure showed that IHHNV Texas and Florida isolates can conform an icosahedral symmetry, as expected for a parvovirus infectious virion (*26*). Thus, the predicted tertiary structure analysis supports the phylogenetic data that IHHNV Texas and Florida isolates represent an infectious form of the virus and not a type 3 strain.

Although it might seem redundant at first sight to perform protein modeling, we considered it fundamental for the study to confirm the capsid protein coding regions were not endogenous viral elements (EVEs). These EVEs are well characterized in *P. monodon* shrimp, but they have not been reported as frequently in *P. vannamei* shrimp. Furthermore, the EVEs from *P. vannamei* shrimp might contain regions of the IHHNV genome that are different to the regions reported in *P. monodon* shrimp*.* Because of the lack of frozen shrimp samples, we could not delineate the pathogenicity of the virus in experimental bioassay, and hence could not perform histopathology to assess cellular manifestation of the viral infection; the in silico analysis of IHHNV capsid protein data gave further confidence that these strains represented an infectious form of IHHNV and not an EVE. A combination of protein modeling, whole-genome sequencing, PCR, and phylogenetic analysis are complementary in nature and sufficient to deem the IHHNV strain detected in the United States in 2019 an infectious type 2 strain of the virus.

IHHNV is well known to be endemic in shrimp-producing countries in the Americas, Asia, and Australia (*7*). IHHNV is also well established in wild shrimp populations in the Gulf of Mexico (*21*). In the United States, however, the virus had not been reported in farmed shrimp since 1993 (*27*). Over the past 4 decades, as shrimp farming evolved from subsistence levels of farming to an intensive culture system worldwide, infectious diseases have emerged and spread regularly. The spread of diseases has been further exacerbated by the movement of broodstock and post-larvae across countries and continents. The movement of infected shrimp has played a critical role in the spread of pathogens like white spot syndrome virus, Taura syndrome virus, and IHHNV (*34*). The origin of the IHHNV isolates described in this study remains unknown, but the data suggest these isolates could have originated in Latin America. This finding highlights the need to keep following strict biosecurity protocols, including disease surveillance in shrimp hatcheries and grow-out ponds, to prevent further introduction of IHHNV or other OIE-listed and nonlisted pathogens in shrimp facilities in the United States and elsewhere.
